# Productivity, resource efficiency and financial savings: An investigation of the current capabilities and potential of South Australian home food gardens

**DOI:** 10.1371/journal.pone.0230232

**Published:** 2020-04-14

**Authors:** Georgia Csortan, James Ward, Philip Roetman

**Affiliations:** School of Natural & Built Environments, University of South Australia, Adelaide, South Australia, Australia; University of Miami, UNITED STATES

## Abstract

As the dominant form of urban agriculture (UA) in Australia, existing home food gardens potentially represent a significant resource in the context of future urban food security and sustainability. However, a severe lack of in-field data has hindered our understanding of the form and function of home food gardens which in turn may hinder innovation and improvement. We investigated the productivity, resource efficiency and potential financial savings of home food gardens in South Australia. A group of 34 citizen science participants measured and recorded inputs and outputs from their gardens. Inputs included time spent on various gardening activities, financial costs, and water use. Outputs included crop yields, from which retail value and nutritional content were then derived. The paper outlines a field-demonstrated, comprehensive methodology for continued and consistent data collection for all forms of UA. We found smaller gardens to be more intensive than larger gardens, requiring higher inputs, but also returning higher outputs per unit area. Both productivity and resource efficiency varied among the gardens, and labour requirements were significantly lower than previously estimated. Water use efficiency of the gardens were calculated and found to have comparable water use efficiency to commercial horticulture. Of the gardens involved, we calculated that 65% should break even in five or less years and save money. After applying a minimum wage almost one in five gardens were financially viable. The results represent the most comprehensive measurements on home food gardens to date, and allow practical, evidence-based recommendations for diversification, time saving and smart irrigation practices to improve garden productivity and enhance the viability of UA.

## Introduction

Anticipation in the potential of urban agriculture is intensifying as the global human population continues to grow, and we face the challenges of feeding more people with limited natural resources. Urban agriculture (UA) is an integral element in our collective vision of a sustainable urban future [1–4], yet its potential contribution to sustainability and food security is poorly quantified. Early research into the economic value of home vegetable gardens in the USA involved small, detailed studies on one or two purpose built, experimental gardens [5–8]. More recent UA research has expanded its focus to typically investigate the yields and retail value of food produced by larger numbers of home and community food gardens [9–13]. Yet this expansion of garden numbers and types also appears to have resulted in less practical garden detail being collected. Of these more recent studies, labour and costs (two key considerations in the potential of UA) were only measured by Codyre et al. [10]. Measurements of water use in urban food gardens, in particular, have been severely overlooked and understudied [14, 15], to the point where our recent PRISMA systematic review found, *“…no recorded attempt to collect any ‘water use efficiency’ data on existing urban food gardens*, *such as those created and managed by home gardeners”*–pg. 7 [16].

Thus, there remains a considerable lack of knowledge regarding many of the practicalities of UA, and several demands for an increase in field-based data collection have been made [17–21]. Without such field-based data on all the key inputs and outputs of urban food gardens, there is no consistent, comparable knowledge on which to base sustainable improvements or to help upscale UA for a greater future contribution to urban food security. At the local level, both households and local government are interested in whether growing food can help people to save money–thereby reducing their cost of living. Home food gardens are the most common form of urban food production in Australia [19] and yet their productivity, resource efficiency and potential financial savings are still relatively unknown. We have a clear need to better understand UA as it currently exists in our urban areas.

The work presented here is the culmination of a large study on the productivity, resource efficiency (including water use and financial sustainability) and social value of urban food gardens in South Australia. Previously published papers drawing on project data include a review of historic and current approaches to measuring UA productivity [22]; a PRISMA systematic review into the water use and water use efficiency of UA [16]; and papers on home food garden diversity [23]; and on the motivations, values, social value, food preservation and food distribution of gardeners [4].

This paper presents the field-collected data from “*Edible Gardens*”, a state-wide citizen science project of the University of South Australia. The project incorporated a citizen science approach as an effective way to overcome many of the challenges inherent in practical UA research, thus allowing the focus to be on the performance of real-world, existing food gardens and gardeners [11, 12, 22]. This paper documents a new and replicable research methodology for collecting input and output data on existing urban food gardens, with an emphasis on home food gardens. Diverse forms of food garden activity, in addition to vegetable and herb production, are considered by these methods, including orchard production and the keeping of urban livestock. The aim of this research was to provide deeper insight into the productivity, resource efficiency and potential financial savings of home food gardens, using South Australia as a case study. Productivity and resource efficiency results are reported, in addition to estimates of return on labour and financial investment, and water use efficiency results for existing home food gardens. We were also able to examine the influence of scale in home food gardens and its potential relationship with intensity of production and resource requirements. Implications of these findings include new ways for gardeners to choose suitable gardening setups, and a practical, evidence-based rationale for improving and optimising UA towards broader goals of urban sustainability, accessibility and food security.

## Methods

### Study location

This research was conducted in the Australian state of South Australia (pop. 1.7 million people [24]), with the capital city of Adelaide (latitude: -34.928, longitude: 138.599). Along the southern coast where most of the population live, South Australia has a mixture of warm to hot dry summers and cold, wet winters [25]. Adelaide itself receives an average of 545 mm of rainfall per annum [26]. The availability of water is a persistent issue, particularly during summer when the evapotranspiration rates are higher than the low rainfall [27].

The Edible Gardens project ran from the end of 2016 until mid-2018. Ethical approval was granted by the University of South Australia–Human Research Ethics Committee in January 2015 (Protocol number 0000034940). The project had two parts, the first an online survey and the second a period of field-based garden data collection by selected “citizen scientists” (members of the public who volunteered and received guidance on how to collect input and output data from their own food gardens). All forms of urban food production were considered, from growing fresh produce in different ways to the keeping of urban livestock such as poultry, bees or fish. The project was open and promoted to home, community and school gardens across South Australia using a combination of online channels and print materials. The online survey was open to South Australian residents over the age of 18. The in-depth survey collected a variety of base garden data, estimates of resource use and insights into food garden experiences [4, 22, 23]. From September 2016 until April 2018, 402 South Australian residents completed the survey.

As part of the online survey, 232 respondents (58%) volunteered to conduct field-based garden data collection. A sampling framework was designed to select participants to ensure the full range of available garden types to those surveyed. Primary selection criteria included: garden size, production methods, estimated inputs (time and expenses), water sources and irrigation methods. Secondary criteria related to gardener details, including gardening experience, gardening consistency, age, education and gender. Following the selection process, 105 gardeners were selected to register their gardens and collect data on their own food gardens. It should be noted that, while the Edible Gardens project results are potentially representative of the different types of food gardens prevalent in South Australia, there are limitations in extrapolating the results to the wider population.

Home gardeners showed the most interest in the project. Of the gardens selected, 71 home gardeners and 2 school gardeners proceeded to register with the project and request data collection toolkits. Although 20 community gardeners completed the online survey and 13 volunteered for field-based garden data collection, none registered to collect data (time commitments and the need to form working relationships with the managing groups of each community garden were problematic).

Registration for field-based garden data collection was done online and involved nominating the number of garden “areas” for which the gardener was willing to collect data (maximum of four areas per garden), uploading a photo and describing the food garden. For each nominated garden area, participants reported its size (area under production), cultivation technique, typical crop/s, water source/s, irrigation method/s, and position on the land block. As a way to distinguish between different individual gardens areas within the registered gardens, “method-crop” categories were developed. The method-crop categories were a combination of the type of production method (or garden area) and the type of typical crop. These categories were developed as a compromise between aggregating data at the level of garden area (not sufficiently detailed), and differentiating data by individual crop (too impractical and time-consuming for participants).

### Data collection

Registered gardeners were posted a data collection toolkit (as reported in [22]) which was customised to suit their garden. The water sources and irrigation methods used dictated what type of, and how many, water meters were supplied to the participant. Two types of water meters were available, positive displacement Elster Model v100 meters (suitable for lower flow and pressure systems e.g. rainwater tanks, and which were kindly donated by SA Water) and small digital impeller meters (suitable for consistent pressure and flow systems such as reticulated mains water) [7]. The toolkits included instructions, data sheets and measurement tools (spring balance scales and water meters). The instructions and data sheets were based upon the Harvest Count section of the Farming Concrete project developed by Gittleman et al. [12]. The participants were asked to measure five variables on an ongoing basis:

1. Time spent on all food-garden related activities (hours & minutes);
2. Money spent on food-garden related purchases ($AUS);
3. Irrigation applied (mains, rainwater, bore/grey water, using water meters) (litres);
4. Harvested crop yields (using spring balance scales or digital scales) (kilograms); and
5. Food shared with others outside of the household (or school) (kilograms).

With regards to time spent irrigating, participants were instructed to log time spent physically irrigating, turning on or moving watering systems but not the length of time an irrigation system was left running (if the gardener was not gardening for the duration). For areas with completely automated irrigation systems, participants were able to log zero minutes for irrigation. Participants were permitted to continue collecting data for as long as they wished, although longer participation was encouraged. Participants entered their data into the Edible Gardens project online system which contained webpages for garden registration, data entry and data visualisation and interaction [22]. As participants entered data, the data visualisation page automatically generated colourful and interactive graphs of the input and yield results for each garden. Each participant could access, download and save their own data at any time during the project.

Three forms of supplementary data were collected by the research team. First, we tracked the retail value of different fresh foods over the course of the project using data collected from two supermarkets, one national retailer and one state-based retailer. The average retail price from the two supermarkets at the time of reported harvest were applied. Although many of the participants reported using organic growing approaches, certified organic retail prices for food products were considered inappropriate due to the high certification costs paid by certified growers. Second, nutritional information, including energy, protein and percentages of edible parts of each crop were collected from the Food Standards Australia New Zealand (FSANZ) online database—Nutrient Tables for Use in Australia (NUTTAB) [28]. Third, natural rainfall data were collected from the Australian Bureau of Meteorology’s Online Climate Database [29], using rainfall data from the recording stations closest to each garden. The natural rainfall data was added to the measured irrigation data, as recommended by Pollard et al. [7] to calculate total water received by plants, enabling greater comparison of the water use results from this study with future studies in wetter or drier climates.

### Statistical analysis

The dataset was cleaned and managed by the research team (the authors). Registered gardens who lacked record of any of the three inputs (time, money and water) or yield measurements, or had less than 30 data entries, were removed from the dataset. Statistical analyses were conducted using SPSS software (version 25. IBM Corp) and Microsoft Excel. The statistical tests used were: Spearman correlations (r_*s*_); Kruskal-Wallis tests (H) with post hoc Dunn’s test, strength rankings from Rea and Parker [30] and Bonferroni corrections; Wilcoxon-signed rank tests (T); and two-way Chi-square tests (*X*^*2*^).

Owing to the variability in garden size and duration of participation, most of the values in this paper are presented as the value per square metre, per 30 days. Presenting results as values per square metre (or square yard) is typical of UA research. The duration of data collection of past studies has varied considerably yet studies tend not to normalise to a standard length of time. For example, Stall [6] reported a yield of 6.79 kg/m^2^ (from 8 months), Cleveland et al. [5] reported a yield of 1.24 kg/m^2^ (from 2.5 years) and 2.31 kg/m^2^ (from 3 years), Codyre et al. [10] reported a yield of 1.43 kg/m^2^ (from 1 year) and Zainuddin and Mercer [31] reported a yield of 0.35 kg/m^2^ (from 3 months). This variability makes comparisons among results challenging. Introducing time as an additional denominator, while also considering the seasonal time of year, allows for clearer comparisons than currently possible, among studies of different durations.

## Results and discussion

Data were collected across South Australia on 34 home gardens, containing 93 individual garden areas. Data collection occurred during the period from November 2016 to June 2018, and within all four seasons, although not all gardens collected data for the entire period. Across all 34 participating gardens, the median duration of data collection was 176 days (just under 6 months), while the total data collection period amounted to 7,565 individual “garden-days” or 252 “garden-months”. The total combined area under production was 3,161 m^2^ and the total harvested yield was 3,479kg of fresh vegetables, fruits, herbs, eggs, fish and honey.

Of the 11 method-crop categories involved in the Edible Gardens project, five had sufficient data (>500 entries) or sufficient numbers of individual areas (>10 across the 34 participating gardens) to form comparable samples. For a summary of all the project data please refer to S1 Table. S2 Table presents an overview of the five dominant method-crop categories, while S3 Table compares the time per activity per method-crop category. Results from all garden areas (and all 11 method-crop categories) are included in the results pertaining to whole gardens. This paper also presents individual results on the five dominant method-crop categories, which were:

1. Bed-orch: In-ground orchard (fruit trees)
2. Bed-mixed: In-ground garden beds producing vegetables, herbs and ‘other’ crops
3. Chkn-egg: Chickens kept for egg production
4. Raised-mixed: Raised garden beds producing vegetables, herbs and ‘other’ crops
5. Wick-mixed: Wicking beds producing vegetables, herbs and ‘other’ crops

There were also two aquaponics systems, totalling more than 430 data entries. However, as there were only two systems, these results were not comparable with the other more dominant method-crop categories and so were not included in the individual method-crop analysis. The method-crop categories support comparisons both between areas within individual gardens and comparisons with other studies. Descriptions on garden types and growing areas have until now had no categorisation system which adequately defined the diversity of existing urban food production. This level of detail is valuable as each of the five main method-crop categories present different input requirements and output returns, which in turn represent various trade-offs.

Of the citizen scientists responsible for the garden data collection, 24 were women and 10 were men, 33 owned (or had mortgages on) their own homes while only one rented. The majority (27) of the participants shared at least some of the food they grew. Hereafter, the Edible Gardens project will be referred to as “EG”.

Combined with the results, the following discussion is split into three parts: (1) new findings on the state of existing urban food gardens (including productivity and resource efficiency); (2) the potential financial sustainability of home food gardens; and (3) implications of these findings, including a new way for gardeners to select the most suitable garden setups for themselves.

### New findings on the state of existing urban food gardens

#### Productivity

**Fig 1:**
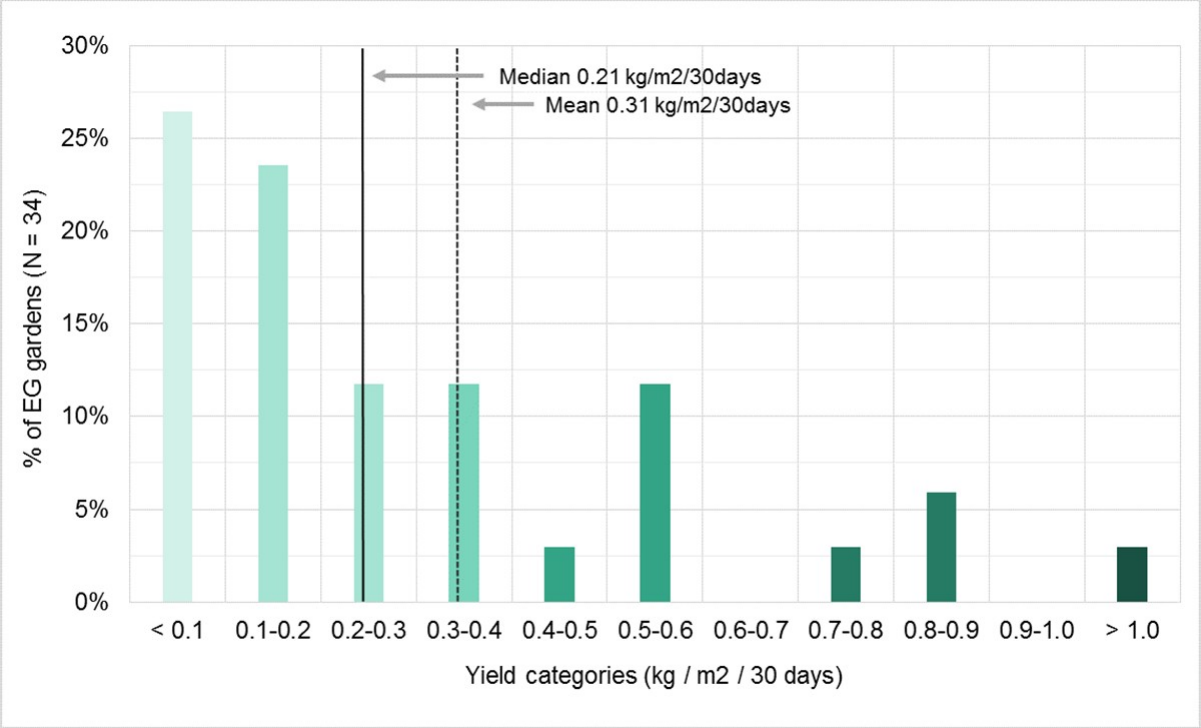
The EG gardens and their yields per square metre per 30 days.

The EG gardens produced a wide variety of vegetables, fruits, herbs and animal products from a range of different cultivation methods. Examples of the most common crops/products were tomatoes, strawberries, parsley and chicken eggs, while some of the more unusual crops included doughnut peaches, Armenian cucumbers, shisho leaves and tromboncinos. For the full list of harvested crops please refer to S4 Table. The total harvested yield of 3,479kg had an estimated supermarket retail value of AUS$28,076. Yields varied considerably among the EG gardens, ranging from 0.02 kg/m^2^/30 days to 1.42 kg/m^2^/30 days. The median yield was 0.21kg/m^2^/30 days. Exactly 50% of the EG gardens produced yields of less than 0.20 kg/m^2^/30 days, while 24% managed to produce higher yields of more than 0.50 kg/m^2^/30 days (Fig 1).

Previous research has also noted the wide variability of yields among individual gardens [10, 11, 13, 20, 31, 32]. Table 1 compares the EG yield results with the yield results of those previous studies which explicitly reported area under production and their duration of data collection. When measuring the year-round inputs and outputs of urban food gardens, time becomes an important consideration to account for natural cycles of more and less productive times of the year. Table 1 also presents the results of this study, both for the combined yield (for all five dominant method-crops) and also for only the vegetable-growing categories, as the previous studies only considered mostly vegetable cultivation.

**Table 1:** Comparison of average yields (kg/m^2^) and (kg/m^2^/30 days) among previous research and the EG results. These results were calculated for the previous studies.

Year	Project name / Authors	No. garden areas (& type)	Location	Reported yield (kg/m^2^)	Average yield (kg/m^2^ /30 days)	Further Details
1979	Stall [6]	1 (created)	Florida, USA	6.79	0.850	Duration: 8 months; Area: 13.9 m^2^; Measured: costs (not include. water), yield & retail value.
1980	Stephens et al. [7]	2 (created)	Florida, USA	2.70	0.450	Duration: 3 months; Area: 189.27 m^2^; Measured: labour, costs, yield & retail value.
1985	Cleveland et al. [5]	2 (created)	Arizona, USA	1.20	0.020	Duration: 2.5 & 3 years; Area: 135.7 m^2^; Measured: labour, costs, water use, yield & retail value.
2014	CoDyre et al. [10]	50 (existing)	Ontario, CAN	1.43	0.006	Duration: 4–5 months; Area: (approx.) 627.5 m^2^; Measured: labour, costs, estimated yield weights & retail value.
2014	Zainuddin & Mercer [31]	15 gardens (existing)	Melbourne, AUS	0.35	0.020	Duration: 3 months by each participant within a 1 year span; Area: 1096 m^2^; Measured: yield & food sharing.
2016	Algert et al. [9]	16 in 8 gardens (created)	California, USA	6.00	0.188	Duration: 17 weeks; Area: 48 m^2^; Measured: yield, retail value & food sharing.
*This study*	*all five main method-crop areas*	82 in 34 gardens (existing)	South Australia, AUS	2.70	0.370	Duration: 1–19 months by each participant within a 19-month span; Area: 3023.3 m^2^; Measured: labour, costs, water use, yield, retail value & food sharing.
*This study*	*only veg/vegh/ herb/other (mixed) areas*	47 in 34 gardens (existing)	South Australia, AUS	3.40	0.470	Duration: 1–19 months by each participant within a 19-month span; Area: 922.3 m^2^; Measured: labour, costs, water use, yield, retail value & food sharing.

While the volume and types of food produced by the different method-crop categories varied, no statistical difference was found among their yields; although the most productive categories (median kg/m^2^/30 days) were raised-mixed, wick-mixed and chkn-egg. Raised-mixed and wick-mixed were also the two method-crop categories with the best median retail value return ($/m^2^/30 days). There was, however, a statistical difference in the retail value returns of the method-crop categories (Kruskal-Wallis test H(4) = 13.04, *p* = 0.0011, Ɛ^2^ = 0.17 (relatively strong)), while the Dunn’s test with Bonferroni correction found that bed-orch had a significantly lower retail value return (*Mdn*. = $0.80) than raised-mixed areas (*Mdn*. = $5.32, *p* = 0.017) and wick-mixed areas (*Mdn*. = $3.48, *p* = 0.044).

**Fig 2:**
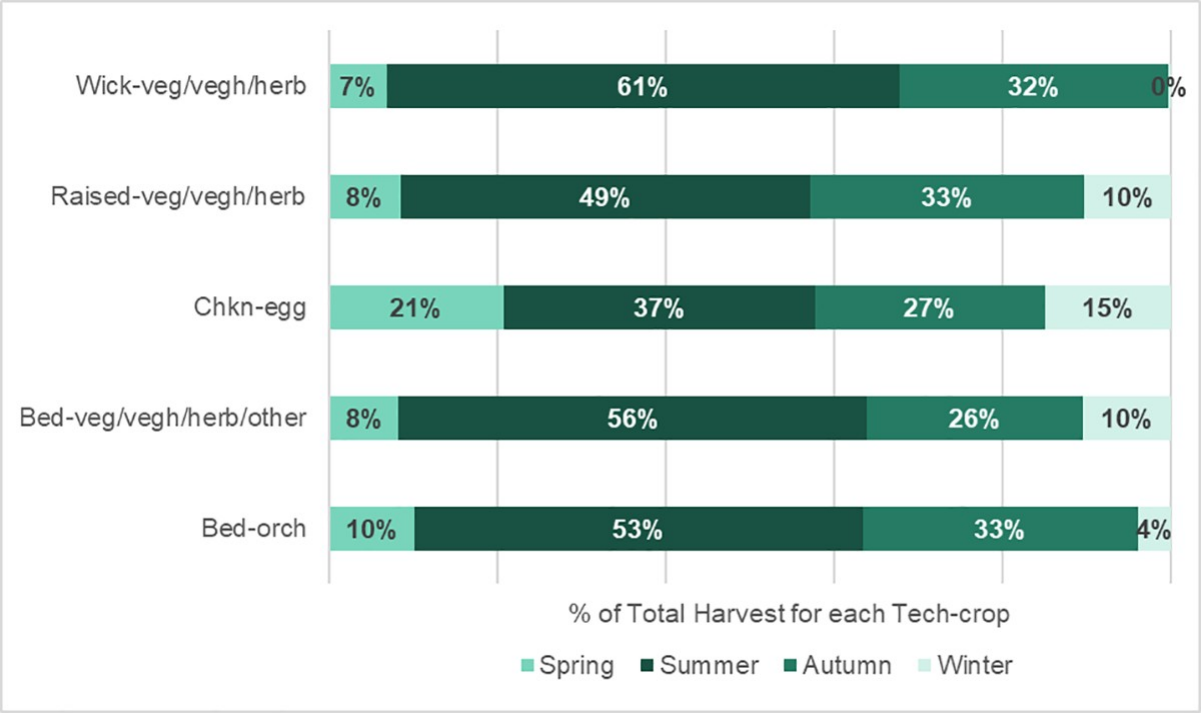
The seasonal proportions of the total harvest quantities from each of the method-crop categories.

Frequency of harvesting was found to differ among the method-crop categories (Two-way Chi-square test X^2^_20_ = 33.60, N = 2646, *p* = 0.029) and by season (X^2^_15_ = 85.80, N = 2646, *p* = 0.001). Looking deeper, there was also a difference when comparing the seasonal harvesting consistency (by number of harvesting events) of each method-crop category (X^2^_12_ = 337.01, N = 4963, *p* = 0.001). Chkn-egg and raised-mixed areas appeared to have the most consistent harvesting event frequency, although this did not translate directly to quantity of food harvested each season (Fig 2).

#### Resource efficiency: Time

Participants tracked all time spent on various food-garden related activities. Table 2 displays the total and percentage times of the different activities. The main activities were: harvesting (31%), irrigating (all kinds combined) (20%), and then livestock care and weeding/pruning (both 8%). ‘Other’ was used as a catch-all for irregular activities including pollination, seed saving, aquaponics system care and repair work. Greater time invested (hours/m^2^/30 days) was positively correlated with greater irrigation (Spearman’s rho r_s_ = 0.570, N = 33, *p* = 0.001) and total water (*W*) (r_s_ = 0.531, N = 33, *p* = 0.001), but greater time also returned higher yields (r_s_ = 0.637, N = 34, *p* = 0.001) and retail value return (r_s_ = 0.738, N = 34, *p* = 0.001). With regards to the dominant method-crop categories, no difference was found among the method-crops and associated time spent on shared activities (two-way Chi-square test *X*^*2*^). As reported previously by Pollard et al. [16] the EG participants used a variety of different irrigation methods to water their gardens. Cleveland et al. [5] estimated spending over 50% of their total time watering their two gardens by hand. We compared this with those EG gardeners who applied water via hand-watering and watering via drippers or sprayers (N = 25) (but not those who utilised automatic irrigation systems). Thus, time spent watering ranged from 13% to 79% of all time recorded, with a median of 37%.

**Table 2:** All monitored garden-related activities and time spent on each^1^.

Activity	Total Labour–all gardens (hours)	Average activity duration (mins)	Median activity duration (mins)	N =	% of total	(rank)
Harvesting	751.4	9	5	34	31%	1
Combined Irrigation	480.8	-	-	-	20%	(2)
*(Mains)*	*246*.*4*	*14*	*10*	*27*	*10%*	*2*
*(Rainwater)*	*214*.*9*	*20*	*12*	*19*	*9%*	*4*
*(Bore/Grey)*	*19*.*5*	*9*	*5*	*5*	*1%*	*13*
‘Other’	223.3	28	5	14	9%	3
Livestock Care	189.3	18	10	18	8%	5
Weeding/Pruning	207.4	50	24	28	8%	6
Plant/Sowing	173.5	54	30	31	7%	7
Soil Prep/Mulch	166.7	70	45	27	7%	8
Building	147.9	123	60	18	6%	9
Sharing Produce	56.4	10	5	27	2%	10
Fertilizing	36.3	29	10	19	1%	11
Pest Control	24.4	50	20	9	1%	12
Total	2,457 hours					

^1^ Not all the 34 gardens reported all the activities. The ‘Other’ category was used as a catch-all for activities which did not fall easily into the prescribed activities, for example, pollinating plants, repairing storm damage, working on aquaponics systems (including feeding fish), and seed saving.

One surprising result was our gardeners’ perceptions that they spent considerably more time tending their food gardens (according to the online survey results [23]) than they reported when physically monitoring their time. Participants’ records of garden labour from in-field garden data collection was compared with their estimation of garden labour (provided for the online survey) using a Wilcoxon signed-rank test (T). The weekly time the EG gardeners estimated in the online survey was significantly higher (*Mdn* = 3.6 hours) than their reported typical weekly hours spent (*Mdn* = 1.3 hours), (T = 22.5, *p* = 0.001, r = 0.8, N = 34). This perception corresponds with findings by Wise [19], who suggested that the estimated time from their surveyed gardeners was considerably higher than the time requirements suggested by professional gardeners. One reason for this difference is that time spent thinking, planning or deciding what to grow, may not have been fully accounted for in the EG in-field measurements.

This finding is particularly interesting when considering that ‘not having enough time’ was both the top initial and the top ongoing challenge faced by the EG survey respondents [23]. Bed-orch areas had the lowest median time requirements (4 mins/m^2^/30 days), followed by chkn-egg areas (8 mins/m^2^/30 days) and bed-mixed areas (9 mins/m^2^/30 days). Overall, the EG gardens had an average labour input of 14 mins/m^2^/30 days. This result is considerably lower than the reported average labour input of the 50 urban food gardens involved in the study by Codyre et al. [10], which equated to 41 mins/m^2^/30 days. This variation could be partially due to the different durations of data collection, with some of EG gardens collecting data for more than a full year, compared with the 4–5 months of growing season suitable for data collection in the study by Codyre et al. [10].

#### Resource efficiency: Area

**Fig 3:**
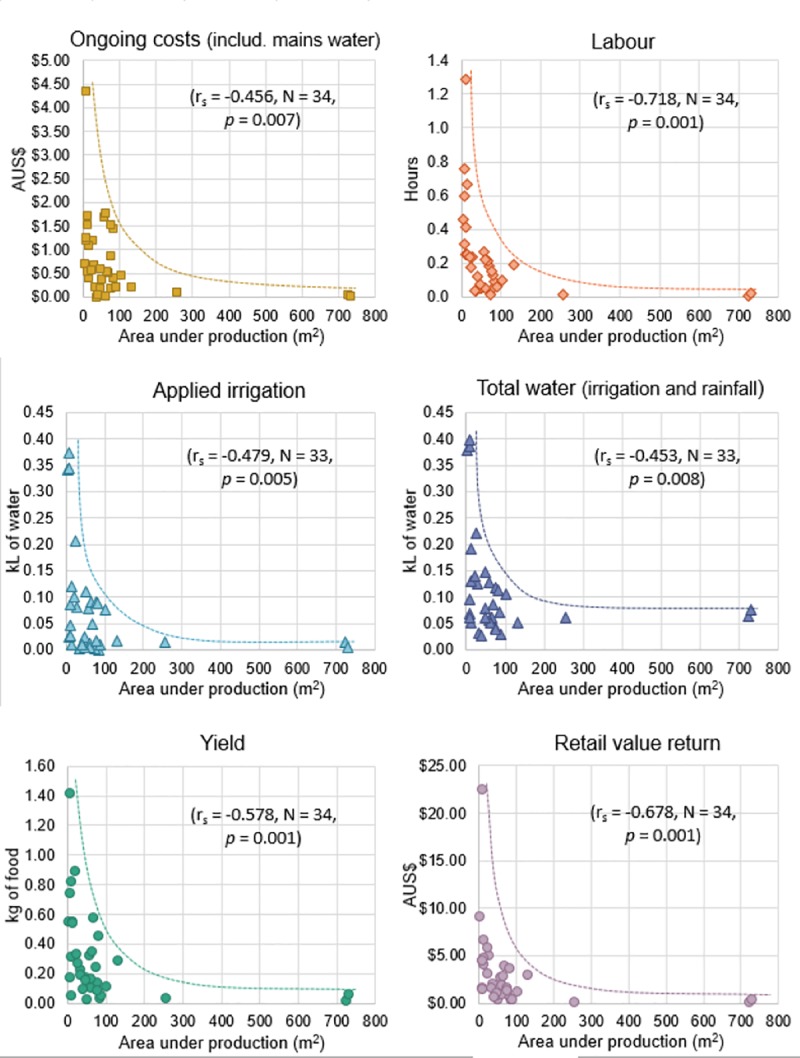
Scatter plots of the negative correlation between area under production and the inputs and outputs. All values are presented as per square metre per 30 days. The lines represent the upper envelope of the results.

Many of the results presented here relate to scale. Of the 34 gardens involved, their total area under production ranged from 4 m^2^ (smallest) through to 731 m^2^ (largest), with a median size of 49 m^2^. Lack of space did not necessarily equal less productive potential. In fact, as the area under production increases, both the inputs and outputs per unit area decrease–i.e. the intensity of the gardening operation effectively decreases with scale. These negative correlations (Spearman’s rho) are displayed in Fig 3, where the lines display an approximate upper envelope as dictated by available data.

This finding corroborates the theoretical findings of a linear programming model by Ward and Symons [33]. They found that per person, smaller gardens of between 10–20 m^2^ were optimal in their ability to produce both high-value and low-water use crops [33]. These results also support approaches recommended by popular gardening literature which try to maximise the use of small spaces close to home, from compact crop varieties, intensive planting, vertical growing structures [34].

Garden size categories were applied to the 34 EG gardens to test for differences: <20 m^2^ (n = 9), 20–50 m^2^ (n = 8), 50–100 m^2^ (n = 12), 100–800 m^2^ (n = 5). No statistical difference among the garden size categories was found in relation to applied irrigation (kL/m^2^/30 days), total water (kL/m^2^/30 days), gardener experience or gardening consistency. Significant differences were found among the ongoing costs, hours spent, yield, retail value and the number of social learning sources utilised by each of the gardeners (Table 3). Ongoing costs were any and all food garden related costs (except setup costs), paid by the EG participants during their data collection periods. The statistically significant results from the Kruskal-Wallis tests and post hoc Dunn’s test with Bonferroni correction are outlined below (Table 3).

**Table 3:** Significant results from comparisons among the garden size categories.

Variable (m^2^/30 days)	Kruskal-Wallis test			Dunn’s test with Bonferroni correction							
	H (df)	*p*	Strength ranking Ɛ^2^	< 20 m^2^		20–50 m^2^		50–100 m^2^		100–800 m^2^	
				*Mdn*.	*Diff*.	*Mdn*.	*p*	*Mdn*.	*p*	*Mdn*.	*p*
**Ongoing costs AUS$**	11.38 (3)	0.010	0.34 (relatively strong)	$1.20	>		--		--	$0.11	0.011
**Labour (hours)**	19.41 (3)	0.001	0.59 (strong)	0.46	>	0.10	0.006	0.11	0.003	0.02	0.001
**Yield (kg)**	9.19 (3)	0.020	0.30 (relatively strong)	0.55	>		--		--	0.06	0.022
**Retail value return AUS$**	13.35 (3)	0.004	0.41 (strong)	$4.47	>		--	$1.46	0.03	$0.29	0.005
**No. of social learning sources**	12.31 (3)	0.006	0.39 (strong)	1	<	3.5	0.005		--		--

The strength rankings of the Kruskal-Wallis tests are based on Rea and Parker [17].

In the Dunn’s test, “*Diff*.” indicates whether the difference between the garden area size >20 m^2^ was greater than or less than the other garden sizes.

A visible *p* value indicates a significance level < α = 0.5

“--” indicates no significant difference.

Consideration of scale also appears to complement the findings on garden diversification. Having a higher number of combinations of method-crop areas was associated with seasonal distribution of harvests (X^2^_9_ = 415.43, N = 4532, *p* = 0.001) and the diversity of harvested yield categories (fruits, herbs, animal products and vegetables) (X^2^_9_ = 603.82, N = 4521, *p* = 0.001). The EG gardens with four different method-crop areas (the maximum allowed in the study, N = 12, with the median no. garden areas = 3), returned the most evenly proportioned seasonal spread of their harvests. And those gardens with three or four method-crop areas appeared to harvest the most even proportions of fruits, herbs, animal products and vegetables. This concept of using a combination of method-crop areas, smaller and more intensive ones—together with larger less intensive method-crop areas with lower input requirements and concomitantly lower yields, in part validates the concept of ‘concentric zoning’ in permaculture literature [35].

#### Resource efficiency: Water

While water was the most challenging input to measure, the data obtained has justified the effort. The median volume of irrigation applied to the EG food gardens was 20L/m^2^/30 days. For those areas only producing vegetables, herbs and other (mixed), the median volume was over double that, 52L/m^2^/30 days. Of the total 690.8kL of irrigation applied, 71% was reticulated mains water, 30% was collected rainwater and 5% was bore or grey water. Bore water is pumped shallow well water. Natural rainfall on the garden areas constituted between 4% - 94% (*Mdn*. 54%) of the total water (combined applied irrigation and rainfall) for each garden. As one of the only previous studies to measure their water use, Cleveland et al. [5] reported the net return for each dollar spent on water as USD$8.80 and USD$7.75 for their two gardens. Our net return per dollar spent on irrigation (assuming the cost of mains water was applied to all irrigation including rainwater and bore/grey water) was between $-41.53 (with some gardens running at a loss) to $169.95 with a median of $13.46 (N = 32 gardens with irrigation data). Cleveland et al. [5] also reported water to be their single largest expense at almost 30% of their total costs. This differed slightly from the EG results, with irrigation contributing between 3% to 90% of total costs, with a median of 23% (N = 27 gardens with cost data). Surprisingly, the cost of mains water was relatively small in the scheme of the total ongoing garden costs. The cost of the water applied to UA can be difficult to measure, as discussed in detail by [16], since although mains water has a set cost there remains disagreement over the levelized costs of alternative water sources. While no water costs for (collected) rainwater, bore or grey water were recorded, it should be noted that these water sources are not free. Statistically, greater total water (*W*) was positively correlated with greater yields (r_s_ = 0.514, N = 33, *p* = 0.002) and retail value return (r_s_ = 0.535, N = 333, *p* = 0.001).

**Fig 4:**
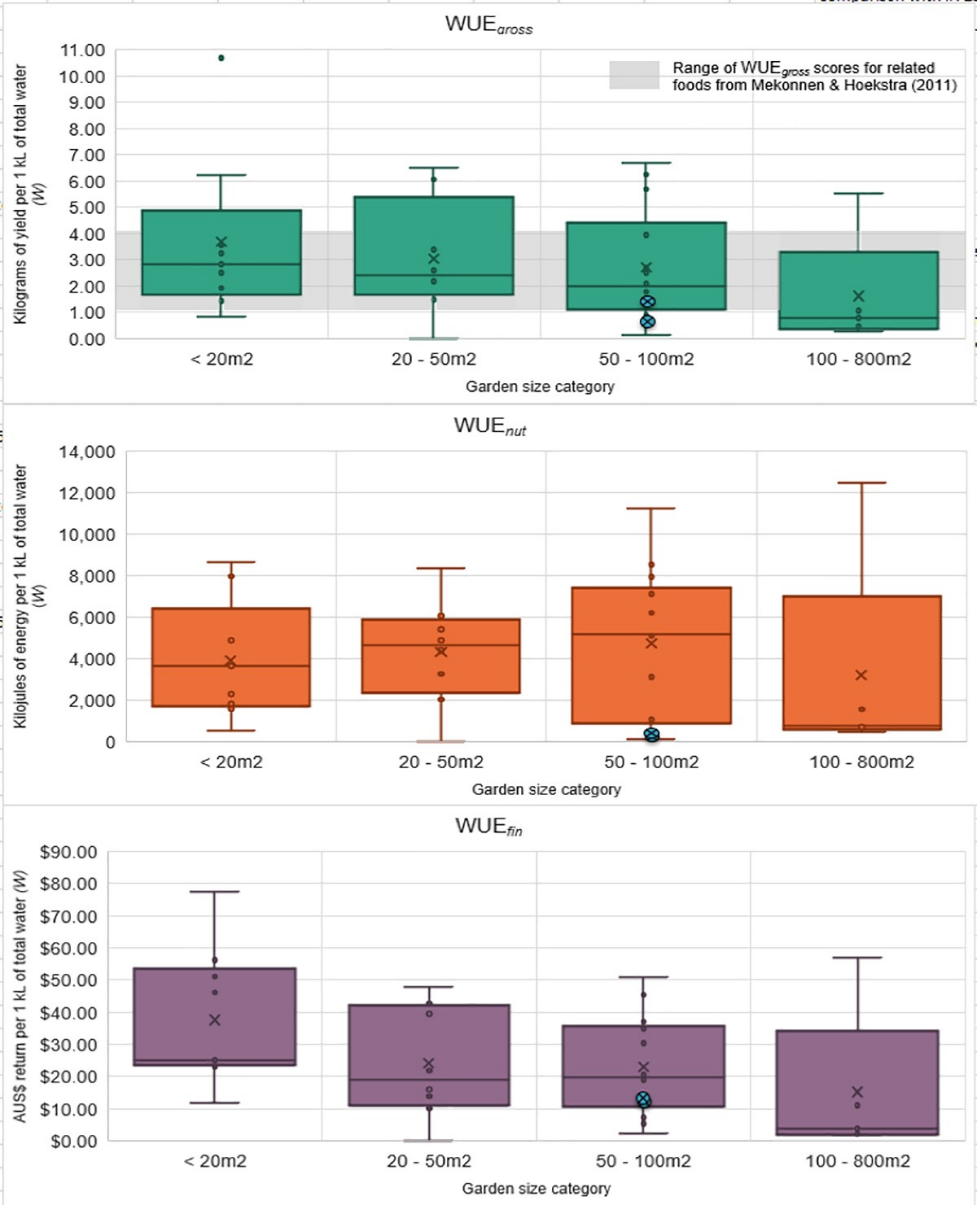
The WUE_*gross*_, WUE_*nut*_ and WUE_*fin*_ results for the garden size categories. The blue circles represent the WUE_*gross*_ and WUE_*nut*_ values of the two gardens from the Cleveland et al. [5] study as previously calculated in [16], while WUE_*fin*_ results have been adjusted to the 2018 AUS$ value. The pale grey band represents the global average WUE_gross_ scores for ‘vegetables’, ‘roots and tubers’ and ‘fruits’ from Mekonnen and Hoekstra [36].

Water use efficiency (WUE) is defined as, “a measure of how efficiently production systems convert water (rainfall and/or irrigation) into a harvestable yield or into money” [16]. Three of the four WUE equations proposed by Pollard et al. [16], were applied to the EG results. The values for simple gross (WUE_*gross*_), nutritional (WUE_*nut*_) and financial (WUE_*fin*_) WUE were calculated for each participating garden (and later for each method-crop category). Fig 4 charts each of the WUE values of the garden size categories. For comparison, Fig 4 also includes the WUE values of Cleveland et al. [5] as previously calculated [16], and the average WUE_*gross*_ of related crops from Mekonnen and Hoekstra [36]. All the EG whole garden WUE scores either matched or surpassed the comparable scores of the two Cleveland gardens.

No statistical difference was found in any of the WUE values and the different garden size categories. However, WUE_*fin*_ was positively correlated with labour (hours/m^2^/30 days) (r_s_ = 0.479, N = 33, *p* = 0.005) and negatively correlated with total area under production (r_s_ = -0.414, N = 33, *p* = 0.017). WUE_*nut*_ was negatively correlated with total water (kL/m^2^/30 days) (r_s_ = -0.438, N = 33, *p* = 0.011).

**Fig 5:**
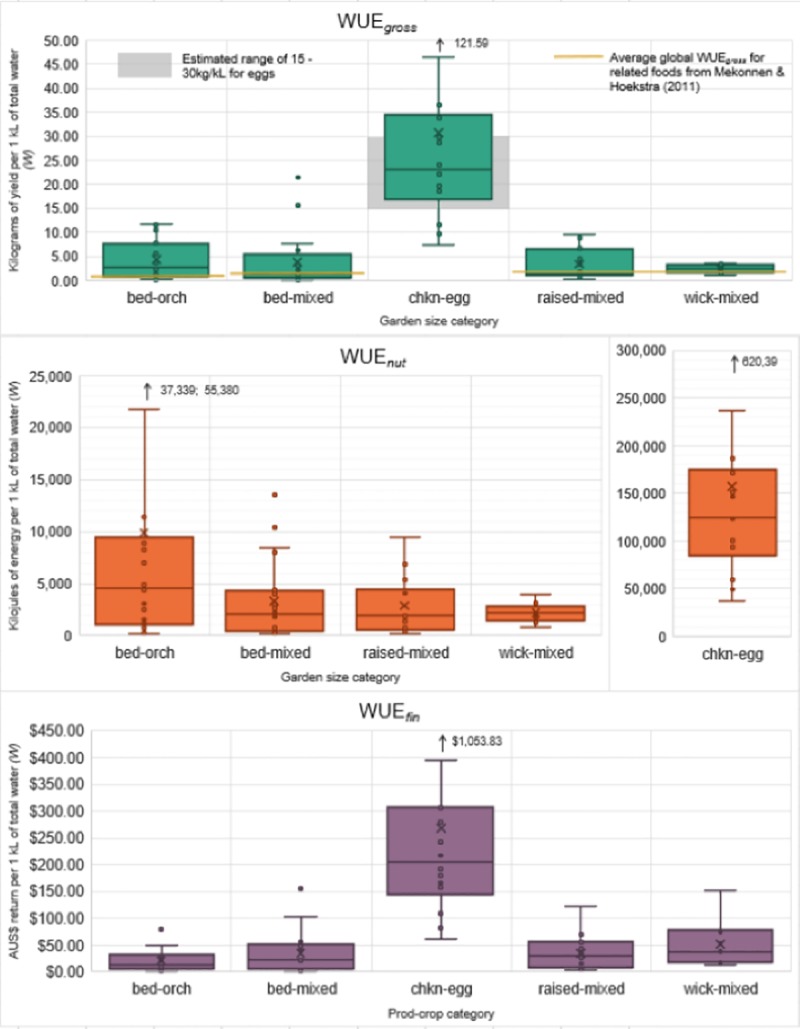
A comparison of the WUE_*gross*_, WUE_*nut*_ and WUE_*fin*_ scores for each of the five main method-crop categories. Outliers are identified by the arrows, and the chkn-egg WUE_*nut*_ scores required its own axis. The additional yellow lines on the WUE_*gross*_ chart represent comparative WUE values of related foods from Mekonnen and Hoekstra [36]. The grey band represents the estimated WUE_*gross*_ range for chicken eggs (irrigation only) as based on a brief review of online poultry keeping websites.

The same three WUE equations were also applied to the method-crop categories (Fig 5) with WUE_*gross*_ compared against global average water footprint of related crops as reported by Mekonnen and Hoekstra [36]. Natural rainfall (also known as “green water”) was not accounted for in the EG calculation of the WUE values for chkn-egg areas, as we assumed that this was not being consumed by the poultry. Comparison with the chicken egg average water footprint reported by Mekonnen and Hoekstra [37] was attempted, however, their blue water figures also account for the irrigation applied to the chicken’s feed. As this study did not consider the embodied water of the feed inputs, chkn-egg areas outcompeted all other method-crop categories in all WUE scores. Although the embodied water in chicken feed is a consideration for broader-scale sustainability, these results are still relevant from an urban water perspective—where keeping chickens for eggs in a home garden is a highly water-efficient option. Thus the WUE_*gross*_ results for chkn-egg areas in Fig 5 are instead compared to a range of estimated WUE_*gross*_ based on a brief review of online poultry keeping websites (assuming between 0.5 – 1L of irrigation per chicken per day, with average of 250 eggs per year at 60 grams per egg, which equates to a range of 15 – 30kg/kL). Wick-mixed presented the lowest variability in both WUE_*gross*_ and WUE_*nut*_, while bed-orch presented the lowest variability in WUE_*fin*_. Bed-orch also presented reasonably strong WUE_*nut*_.

Interestingly, while the lower bounds of the EG method-crop WUE scores matched the average global WUE_*gross*_ values of related crop types from Mekonnen and Hoekstra [36], the majority of the method-crop WUE scores ranged considerably higher. This suggests that the blue and green water footprint of home-produced food (not accounting for chkn-egg areas) can be comparable in water-efficiency to conventional farming, and that the EG participants were generally not substantially over- or under-watering. Chkn-egg areas performed well in all the water-related metrics. They had the lowest median total water (W) requirements (10L/m^2^/30 days), followed by bed-orch areas (51L/m^2^/30 days).

Despite the apparent difference in WUE values, the only statistically significant differences among the method-crop categories were between chkn-egg and the other four main method-crop categories (Table 4). There were differences in WUE_*gross*_ (H(3) = 31.71, *p* = 0.001, Ɛ^2^ = 0.41 (strong)), WUE_*nut*_ (H(3) = 36.40, *p* = 0.001, Ɛ^2^ = 0.47 (strong)) and WUE_*fin*_ (H(3) = 34.69, *p* = 0.001, Ɛ^2^ = 0.45 (strong)).

**Table 4:** Significant results from WUE comparisons among the method-crop categories.

Variable	Dunn’s test with Bonferroni correction									
	Chkn-egg		Bed-orch		Bed-mixed		Raised-mixed		Wick-mixed	
	*Mdn*.	*Diff*.	*Mdn*.	*p*	*Mdn*.	*p*	*Mdn*.	*p*	*Mdn*.	*p*
WUE_*gross*_ (kg/kL)	22.04	>	2.57	0.001	1.10	0.001	1.25	0.001	2.51	0.004
WUE_*nut*_ (kJ/kL)	123,121	>	4,347	0.001	1,813	0.001	1,607	0.001	2,134	0.004
WUE_*fin*_ ($/kL)	192.35	>	10.42	0.001	21.18	0.001	27.52	0.001	38.16	0.046

“*Diff*.” indicates whether the difference between the method-crop category “chkn-egg” was greater than or less than the other categories.

A visible *p* value indicates a significance level < α = 0.5

Another interesting finding was that wick-mixed areas did not outperform the other areas in terms of their applied irrigation or total water (*W*) requirements. A wicking bed is defined as, “… a plant driven system where plants receive water through capillary rise from a self-contained coarse material-filled subsoil reservoir” [29, pg. 1]. Neither did wick-mixed areas present significantly better WUE scores. Indeed, the only metric where the wick-mixed areas out-performed the other categories was with their frequency of irrigation. There was an association between the method-crop categories and the frequency of irrigation (X^2^_12_ = 155.76, N = 1146, *p* = 0.001). The dominant frequencies were weekly and then every three weeks for wick-mix (43%; 30%) and similarly for bed-orch (40%; 33%). For chkn-egg areas the most dominant two frequencies were weekly and then daily (34%; 31%). Whereas the most dominant two frequencies were daily and then weekly for bed-mixed (46%; 31%) and for raised-mixed (50%; 40%). This finding supports the only other published scientific research on wicking beds, by Semananda et al. [38], who found wicking beds to be significantly more labour efficient than precision surface irrigation. While growing seedlings, their experimental wicking beds could go up to 4 weeks without irrigation, and with mature tomato plants could go 1–2 weeks without irrigation [38].

The overall inputs and outputs of each of the main method-crop categories are displayed in Table 5. The method-crop categories with the lowest median input requirements were bed-orch (labour), chkn-egg (total water) and bed-orch (ongoing-costs). The highest median input requirements were wick-mixed (labour), bed-mixed (total water) and raised-mixed (costs). The method-crop categories with the greatest median returns were raised-mixed (yield and retail value), while the lowest median returns were from bed-mixed (yield) and bed-orch (retail value).

**Table 5:** A comparison of the median input, output and WUE results of each of the five main method-crop categories.

Method-crop Category	bed-orch	bed-mixed	chkn-egg	raised-mixed	wick-mixed
Median labour (mins) /per m^2^ / 30 days	**4**	9	8	16	18
Median total water (*W*) (L) / per m^2^ / 30 days	51	99	**9**	93	95
Median irrigation (L) / per m^2^ / 30 days	**5**	21	9	52	52
Median costs (+ mains water)/ per m^2^ / 30 days	**$0.02**	$0.51	$0.77	$0.82	$0.60
Median yield (kg) / per m^2^ / 30 days	0.19	0.17	0.25	**0.39**	0.27
Median retail value AUS$ / per m^2^ / 30 days	$0.85	$2.25	$2.19	**$5.32**	$3.48
Median net position AUS$ / per m^2^ / 30 days	$0.80	$1.61	$1.04	**$2.18**	$1.86
Median WUE_*gross*_ (kg/kL)	2.57	1.10	**22.04**	1.25	2.51
Median WUE_*nut*_ (kJ/kL)	4,347	1,813	**123,121**	1,607	2,134
Median WUE_*fin*_ (AUS$/kL)	$10.42	$21.18	**$192.35**	$27.52	$38.16
**Median size (m**^**2**^**)**	**10.0**	**15.0**	**10.0**	**12.0**	**5.0**

### Financial sustainability

#### Setup costs and ongoing costs

While the online survey did collect information on respondents’ entire food garden setup costs, information was not collected on the setup costs of individual growing areas. The setup costs for the method-crop areas were therefore calculated based on a combination of additional investigation into the typical retail costs of component parts, together with detailed costing obtained (in follow-up communications) from at least five participants per method-crop category. Time requirements and cost of labour were not included in these calculations. Some differences were found among the setup costs for the five main method-crop categories. Bed-mixed had the lowest average setup cost (AUS$18.91/m^2^), followed by bed-orch areas (AUS$22.73/m^2^). Table 6 presents an overview of the various costs, value and the calculated time to break even for each dominant method-crop. For a breakdown of the calculated setup cost details and assumptions, please refer to S5 Table. It should be noted that these costs include the retail costs of purchasing all the separate parts, and these figures could change with the use of second-hand or salvaged materials. Economies of scale have also not been fully considered, and the total setup costs of different areas may change according to total size and intensity of use. This is clearly an area that warrants future research.

**Table 6:** A comparison of the related costs and time required to break even for each of the five main method-crop categories.

Method-crop	Average setup cost ($ per m^2^)	Median monthly costs (+ mains water) ($ per m^2^)	Median monthly retail value ($ per m^2^)	Min–Max (years)	# “never” break even (%)	Median time until break even (years)
Bed-orch	$22.73	$0.02	$0.85	0.42–62.1	3 (15%)	2.0
Bed-mixed	$18.91	$0.51	$2.25	0.04–11.70	4 (17%)	0.5
Chkn-egg	$121.46	$0.62	$1.87	0.39–38.00	3 (19%)	4.6
Raised-mixed	$106.09	$0.74	$5.27	0.91–7.90	5 (28%)	1.1
Wick-mixed	$222.82	$0.60	$3.48	0.76–58.00	1 (11%)	2.0

A series of Kruskal-Wallis tests were used to look for statistical differences among the method-crop categories. The median break-even time for all the categories was less than five years, and although the initial Kruskal-Wallis test returned a significant result with differences among the method-crop categories (H(4) = 11.10, *p* = 0.025, Ɛ^2^ = 0.14, N = 80), the follow on post hoc Dunn’s test with the Bonferroni correction did not identify any significant pairwise comparisons.

Interestingly, unlike the misperception on time spent, when comparing the estimated average monthly garden costs from the initial survey and the average reported monthly garden costs, no significant difference was found. The EG participants were far more accurate estimating their costs than their time. Individually, bed-orch areas again had the lowest median ongoing costs (including the cost of mains water) (AUS$0.02/m^2^/30 days), followed by bed-mixed (AUS$0.51/m^2^/30 days) and wick-mixed (AUS$0.60/ m^2^/30 days). There were significant differences in the ongoing costs and the labour of the method-crop categories, with bed-orch areas having much lower ongoing costs and labour requirements than the other four method-crop categories. The differences in ongoing costs AUS$/m^2^/30 days were (H(4) = 27.24, *p* = 0.001, Ɛ^2^ = 0.347 (relatively strong)) and the differences in labour hours/m^2^/30 days were (H(4) = 21.53, *p* = 0.001, Ɛ^2^ = 0.271 (relatively strong)), with post hoc test results presented in Table 7.

**Table 7:** Results from comparisons in ongoing costs and labour requirements among the method-crop categories.

Variable (m^2^/30 days)	Dunn’s test with Bonferroni correction									
	Bed-orch		Chkn-egg		Bed-mixed		Raised-mixed		Wick-mixed	
	*Mdn*.	*Diff*.	*Mdn*.	*p*	*Mdn*.	*p*	*Mdn*.	*p*	*Mdn*.	*p*
Ongoing costs ($)	0.02	<	0.62	0.003	0.51	0.004	0.74	0.001	0.60	0.004
Labour (mins)	4	<	8	0.021	9	0.018	16	0.001	18	0.007

“*Diff*.” indicates whether the difference between the method-crop category “bed-orch” was greater than or less than the other categories.

A visible *p* value indicates a significance level < α = 0.5

The EG survey respondents reported contributing similar proportions of home-grown food to their households as South Australian households did 40 years ago, according to a comparable question from a 1975 survey by Halkett [39] (Table 8). The main difference between these two sets of results lies in the category ‘Less than 5%’. We expect that this is due to our having two additional response categories, ‘Unsure’ and ‘break even’, both of which are similar to the ‘Less than 5%’ category. Interestingly, the total proportion of respondents confident that they offset a reasonable proportion (>5%) of their fruit and vegetable bill remains similar between the current cohort (38%) and that of the 1975 study (34%). Therein lies a potential opportunity for new approaches or guidance to help increase the productivity and resource efficiency of home food gardens, and thus increase the contribution of home-grown food in South Australia.

**Table 8:** Comparison of the Edible Gardens survey question on households saving money and a similar question from the 1975 Adelaide study by Halkett [39] on household fruit and vegetable requirements.

Edible Gardens Project			Study by Halket (22)		
Percentage of weekly household fruit and vegetable budget	Count	% of total (n = 374)	% of total (n = 430)	Count	Percentage of household fruit and vegetable requirements
None	79	21%	22%	96	None
Less than 5%	52	14%	46%	198	Less than 5%
6–25%	62	17%	21%	91	6–25%
26–75%	64	17%	8%	33	26–75%
Over 75%	14	4%	5%	12	Over 75%
Unsure	55	15%	-	-	No comparable category
Break even	48	13%	-	-	No comparable category

#### “Breaking-even” and returns per hour

**Fig 6:**
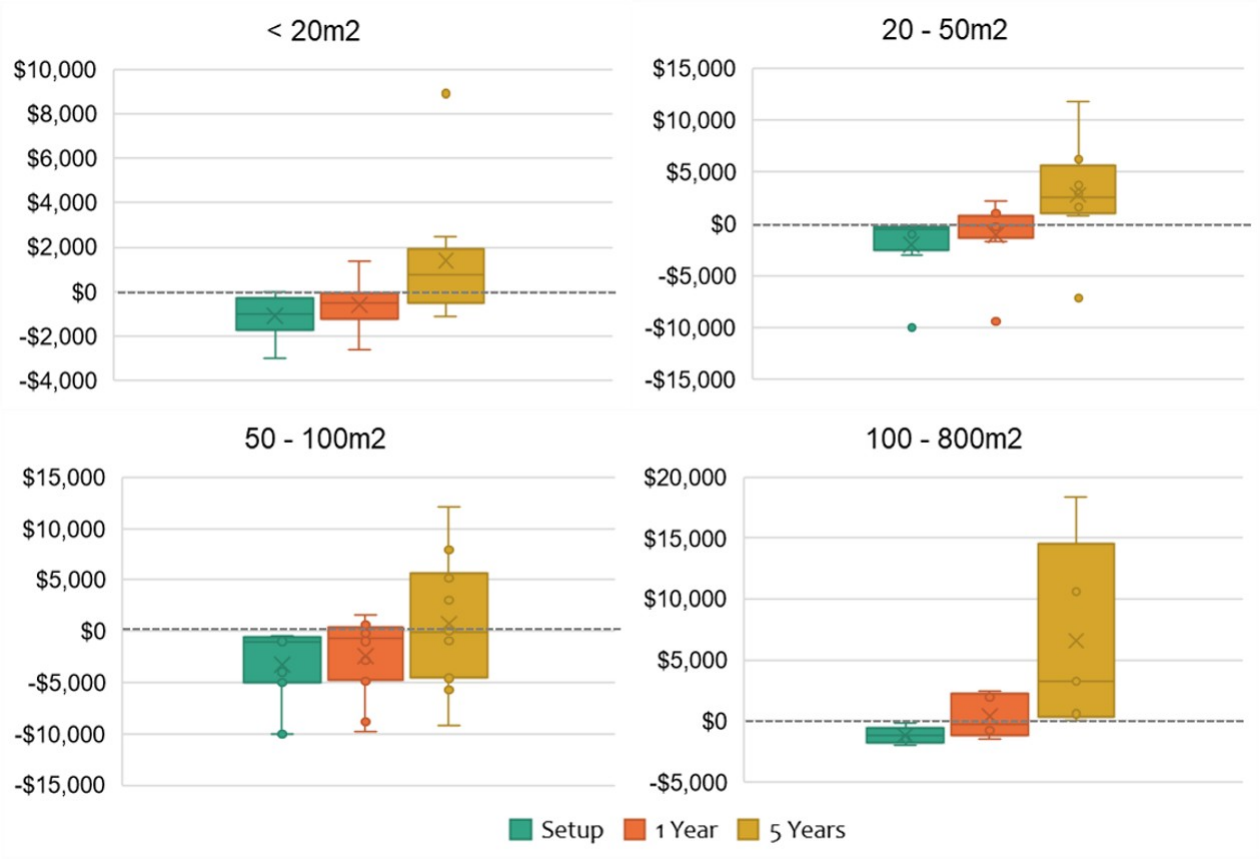
A progression of the setup, 1-year and 5-year net position of the different garden size categories (N = 34).

By considering each garden’s reported setup cost, average monthly cost and average monthly retail value of the food produced, it was possible to calculate the length of time until each entire garden re-couped associated costs and would theoretically “break even”. Note that the setup costs for each garden were based on the gardener’s estimated online survey response. Table 9 displays the length of time to break even for the EG gardens, while Fig 6 displays the setup, one-year and five-year net positions of the four garden size categories.

**Table 9:** Percentage distribution of the calculated time to break even.

Years to break even	No. of gardens	%
< 1 year	10	29
1–5 years	12	35
5–10 years	3	9
> 10 years	3	9
Never (costs greater than retail value return)	6	18
Total:	34	100

**Fig 7:**
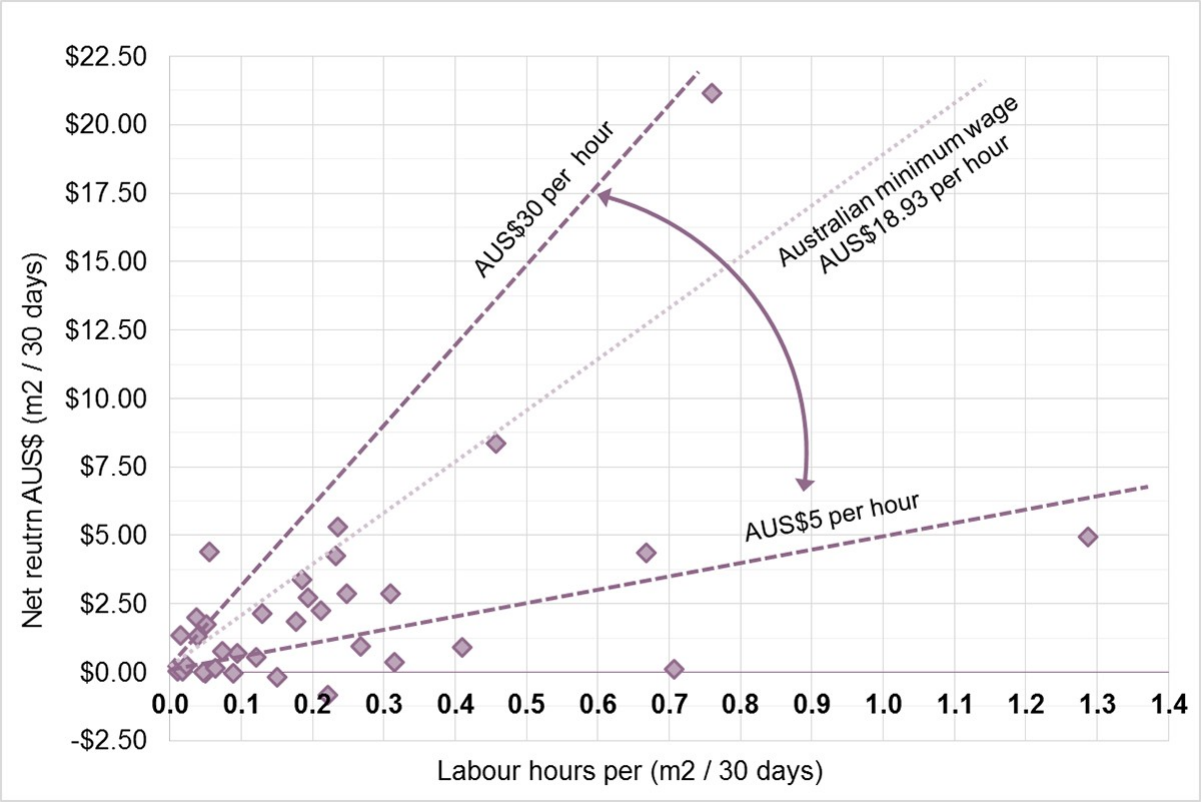
Net return (m^2^/30 days) plotted against labour (m^2^/30 days). Additional lines represent the lower (AUS$5/hour), Australian minimum wage (AUS$18.93) and upper (AUS$30/hour) bounds of the returns for labour.

On a more day-to-day level, the net returns (retail value minus costs including the cost of mains water) per hour invested by the EG participants ranged from AUS-$3.73 to AUS$87.76 with a median of AUS$9.91 (N = 34). Fig 7 displays the full range of net returns and lines for comparison at AUS$5 per hour and AUS$30 per hour net returns. A line indicating the current Australian minimum wage for full-time work (AUS$18.93/hr) has also been included [40].

**Fig 8:**
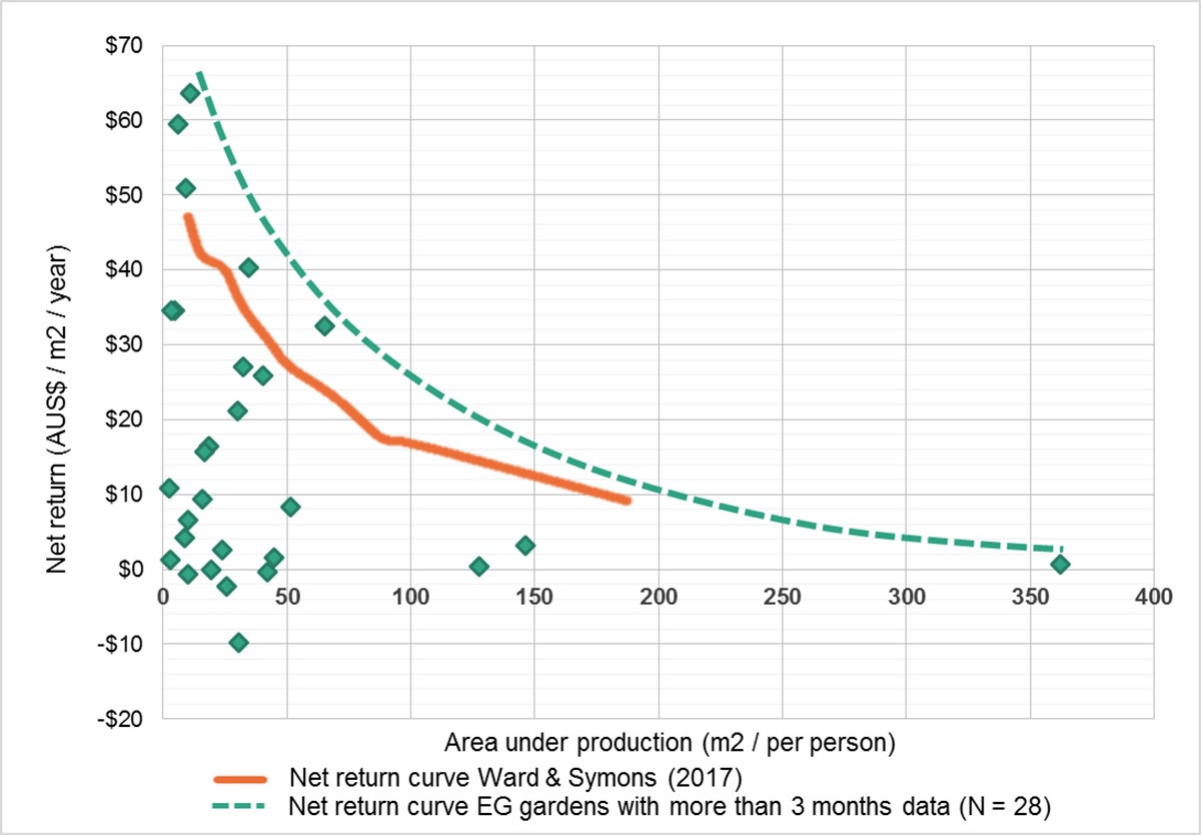
Comparison between the net return curve modelled by Ward & Symons (2017) and the upper net return envelope of those EG gardens with more than 3 months of data (N = 28).

The EG net return and the area under production can also be compared with the net return and garden area per person as modelled by Ward and Symons [33] (Fig 8). Ward and Symons [33] used a two-stage linear programming model to optimise the theoretical net return of UA as based on home food gardens contributing to the dietary requirements of a household. It should be noted that the only inputs costed by Ward and Symons (25) was fertiliser and water, therefore their modelled results may be lower if taking all ongoing costs into account.

Wise [19] hypothesised that approximately 16% of Australian food gardeners were producing yields worth more than AUS$250 per year and thus achieving reasonable financial savings. A more recent study by Algert et al. [9] investigated the potential cost savings and nutritional improvements of home grown food for low-income households. Algert et al. [9] found that their eight home gardeners saved an average of USD$339 over the 4-month summer season. Comparing results is challenging as their gardeners’ ongoing costs were estimated but not explicitly reported, nor was any labour data recorded. Whereas Codyre et al. [10] reported an average cost of USD$10.82/kg, compared with the AUS$8.07/kg of the EG gardens. The EG garden data reveals slightly different results on how many gardeners were set to save money. Even when taking the ongoing costs of each garden into account (but not the initial setup costs), 79% of the 34 EG gardens were calculated to save an excess of more than AUS$250 per year. The numbers were verified by a subset of long-term participants who had collected more than 240 days of data (N = 14), with 71% of these saving an excess of more than AUS$250 per year. While this finding is quite positive for the large numbers of households looking to save money, the full cost of setting up a food garden has not yet been considered.

According to combined participant and author calculations of setup costs, 65% of the EG gardens would overcome both their initial setup and ongoing costs and break even within five or less years. Assuming garden assets are relatively long-lived (much longer than five years), after the setup costs have been paid off, it should be easier to gain reasonable savings for a considerable time. This calculation was based on the average retail value of the food produced by the EG gardens, according to prices from two supermarket retailers. The time to break even would be considerably shortened if the calculation was instead based upon the retail value of certified organic produce as sold at supermarkets. Indeed, a brief comparison of 58 fruits and vegetables from two supermarket retailers (prices collected August 2019) found the certified organic prices to be on average 93% higher. In Australia, the price of organic foods is reported as the main barrier to purchasing [41]. Thus, for those households desiring to eat organic food but struggling to afford the higher prices, producing their own food as organically as possible is one way to access a cheaper alternative.

The median return rate on labour invested in the gardens was AUS$9.91 per hour. If gardening is taken as a simple hobby, with time spent as discretionary then the median hourly return could be considered reasonable. Alternatively, for people who classify themselves as ‘under employed’ (defined as those who wish to work more, and are available to work more paid hours but who currently work less than 35 hours per week [42]), the labour data on the time requirements of different food garden related activities can help to account for the opportunity cost for someone considering whether to grow food as a form of alternative or supplementary employment. As of late 2018, South Australia had an unemployment rate of 5.2%, an underemployment rate of 9.6% and a youth unemployment rate of 16.5% [43], thus this is clearly an area which would benefit from closer study.

#### Impact of applying a wage rate

Comparatively, applying a fixed wage rate (such as minimum wage) to the labour required to produce food, and adding it to the input costs, substantially alters the financial viability of the EG gardens. If gardens can only reliably deliver produce by valuing labour at less than minimum wage, then this needs to be considered when evaluating any widespread contribution to future food security. Moreover, the cost of labour becomes a critical factor when considering the viability of potential commercial UA opportunities. One of the early studies from 1980 by Stephens et al. [7] found that even when the minimum wage of the time (USD$3.10/hr) was applied, the two gardens in their study both still returned net profits. It is interesting to note, however, that even then the labour costs consumed 19–51% of the retail value of the harvested crops and made up between 50–72% of the total garden costs, and the cost of urban water–for example–has risen considerably since 1980. In a more recent study, even before accounting for labour, the gardens involved in CoDyre et al.’s [10] study were already calculated to run at a loss. The retail value of their harvests was based on the average price of 1kg of mixed vegetables with the same crop proportions as the average harvest. This resulted in an average value of USD$4.58/kg. Once the minimum wage rate of the area was applied, labour costs constituted 70% of their total costs and were more than double the other ongoing costs of the gardens [15].

**Fig 9:**
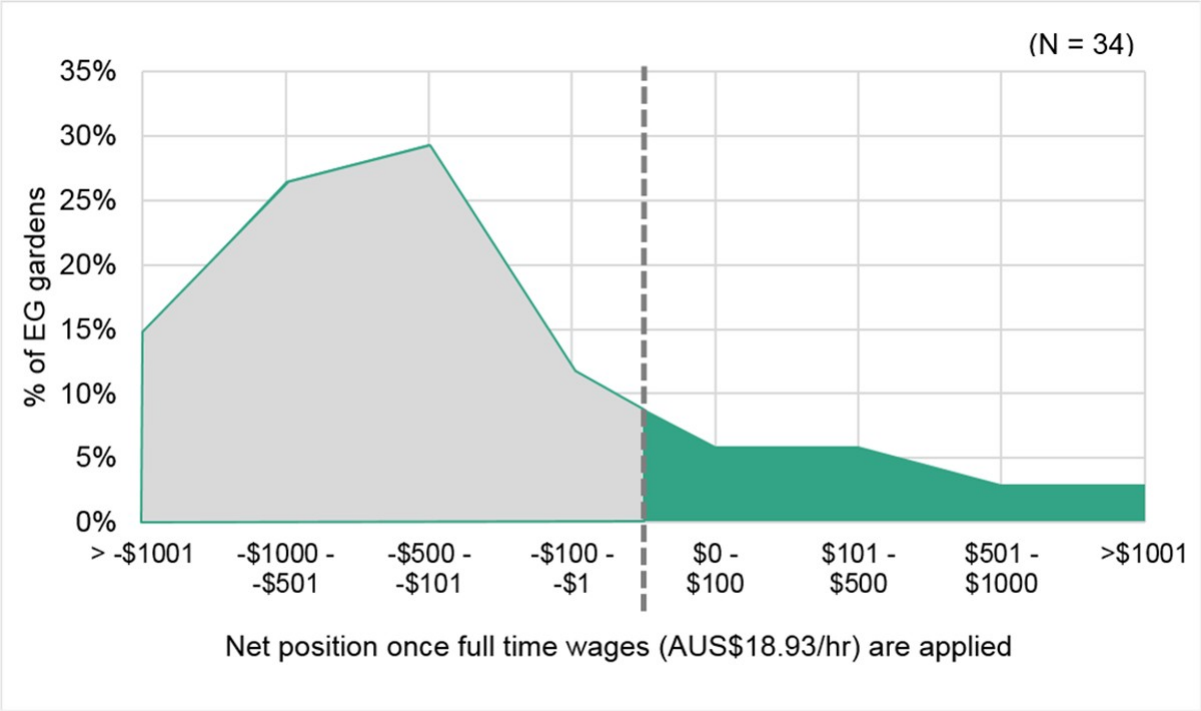
The impact of applying a wage rate to invested labour and how this impacts the net position of the EG gardens.

The EG gardens median return rate of AUS$9.91 per hour is approximately half of the current Australian minimum wage for full time work (AUS$18.93 per hour) [40]. When applying AUS$18.93 to the labour invested by the EG gardeners, the majority (82%) of gardens were in a net loss position (Fig 9) on an ongoing basis, even without taking the initial setup cost into account.

However, even once the minimum wage rate was applied, 18% of the EG gardens did produce enough food to effectively pay for their time at a legal wage rate. A further 12% of the EG gardens were out of pocket, but by less than AUS$100, thus it could be argued that it would not take much of either an increase in productivity or a reduction in labour, to shift those gardens into a positive net position. It is important to note that these results are based on our monitoring and assessment of the relatively simple forms of urban food production involved in the EG project, i.e. methods commonly utilising hand tools and manual irrigation. A commercial urban food business may be organised differently and could potentially use a combination of labour-saving techniques and/or tools. Yet, the wage rate is likely become one of, if not the greatest, expense for commercial UA–even without considering the possibility of casual labour rates (an additional loading of 25% in Australia [40]), or whether a UA business is able to sell their produce at retail or wholesale prices. This is an important consideration as new UA businesses are more likely to arise from serious home food gardens, rather than from any downscaling by commercial horticulturalists or farmers.

### Implications of the findings

To frame these results in a practical way the following section presents a series of recommendations, questions and comparative scenarios. To begin with, we recommend current and future food gardeners ask themselves two basic questions. The first is, “What do you want to get out of growing food?” This question relates to previously presented results from the online survey questions asking people about their key motivations [4]. The top five motivations of home gardeners were: 1) Producing fresh tasty produce, 2) Enjoyment, 3) Health reasons, 4) Natural connection and 5) To save money [4]. Indeed, saving money was a motivation for 59% of the EG survey respondents. The second question is, “What are your biggest challenges or limitations?” This question relates to our previously presented challenges [23]. The top six initial challenges for home gardeners when they first began producing food were: 1) Lack of time, 2) Unsuitable conditions, 3) Lack of knowledge, 4) Lack of space, 5) Cost and 6) Water issues [23]. Both the motivations and challenges of an individual gardener (or household) can be used to help guide their choices in determining which method-crop category (or combination of categories) would best suit them.

**Fig 10:**
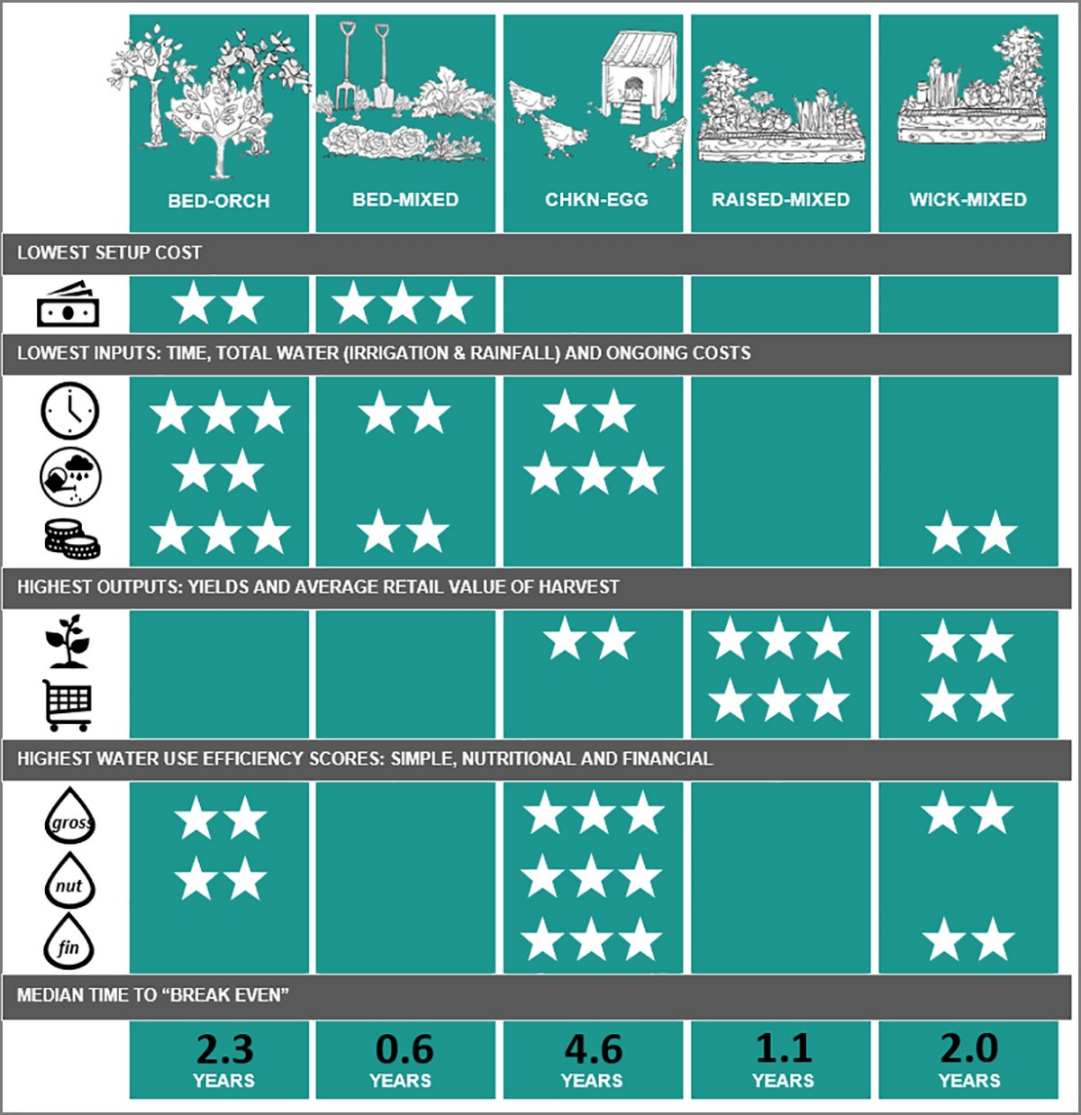
Comparison and ranking of the five main method-crop categories by their inputs and outputs per square metre per 30 days. The number of stars represent the best performers in that category.

A similar perspective was investigated in a Tasmanian study by Kirkpatrick and Davison [21], who surveyed whether garden setups, gardener practices and gardener motivations were statistically related to each other. However, they found only two sets of “garden”, “practice” and “motivation” to have positive associations (5). The approach presented here differs by our attempt to purposefully better relate gardener motivations and challenges to their garden setups from the outset (via the selection of suitable method-crop categories). Fig 10 summarises and ranks the input and output results of the five dominant method-crop categories on a per square metre per 30-day basis.

No single method-crop category presented well across all inputs and outputs. There were different trade-offs for each. It is also important to note that the median size of each category is different–thus changing the results for entire or larger method-crop areas.

#### Recommendations to improve garden results

Gardener experience (as typically measured in years), is an interesting variable. Years of experience has been found to have a positive relationship with gardening efficiency [10], and both a positive [8] and no measurable relationship, with garden productivity [9]. We would argue that both knowledge and skills–and not just the number of years–are needed to apply and learn from food gardening experiences. Using the results from the EG project, we make a series of recommendations which may contribute to food gardener knowledge and thus potentially improve productivity and resource efficiency. This section includes insights into diversification, time saving and smart irrigation practices.

**Fig 11:**
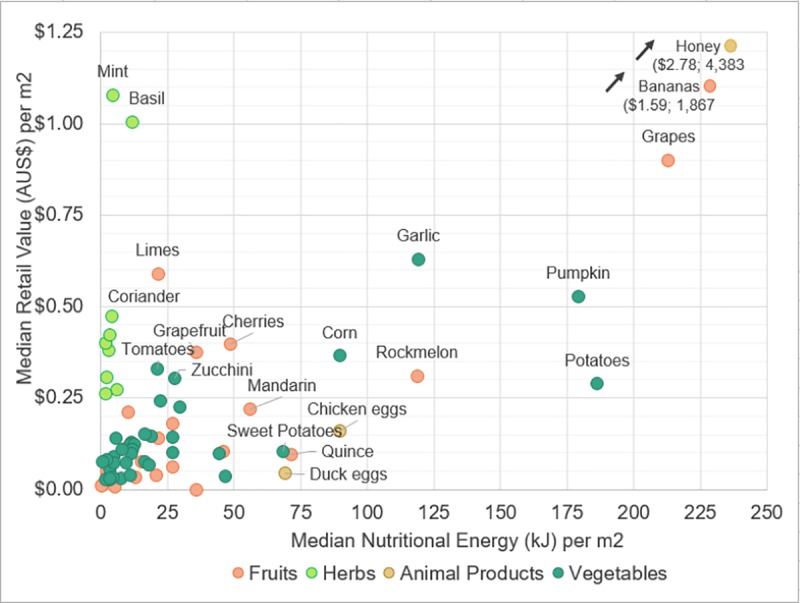
The nutritional content and retail value of the four food types.

As discussed briefly above, some diversification of cultivation techniques may help to even out the input requirements and outputs of a single garden, provide more consistent year-round harvests and produce the most even (and diverse) combination of vegetables, fruits, herbs, and animal products (if desired). This combination of food types can also help balance the nutritional energy content and retail value return of an entire food garden (Fig 11).

In some cases, different techniques can co-exist in the same area (such as chickens under fruit trees). And yet, too many cultivation techniques and gardening approaches in a single garden was found to have a negative relationship with labour, yield and retail value per unit area. Potentially, utilising too many different techniques and approaches results in gardeners spreading themselves too thinly; at least some degree of specialisation is helpful. It has also been previously suggested that some diversification of food production can be more resilient overall in the face of natural disasters or climate-related challenges [44–46]. Diversification can also be implemented via crop type or crop variety choices. One way to both save additional money and to help extend the harvest period is to produce early- and/or late-season crops. Producing crops before or after their peak period, when they are at their most expensive to purchase from supermarkets can provide additional savings. For households who do not want to spend, or do not have the money to spend significant amounts of money setting up new food garden areas, several ways to reduce the initial outlay were reported by the EG survey respondents. Materials or parts can be purchased second-hand, plants can be grown from seed instead of being bought as seedlings, and materials can be salvaged or found for free.

Food gardening does not appear to take as much time as was initially perceived by the participants. But for gardeners who consider themselves “time-poor”, the two main activities to focus on for time-saving are harvesting (31% of all time spent) and irrigating (20% of all time spent). Developing quicker harvesting methods and planning different crops with rapid harvesting in mind can help to reduce labour requirements. It should also be noted that although the bed-orch areas required the lowest amount of ongoing labour, newly planted trees do require time to establish their roots before any reasonable harvest can be expected.

In addition to harvesting, irrigation is a core component of food production. Irrigation took 20% of the total labour of the EG gardens. This time did not account for the durations that irrigation systems were running, only physical time spent watering. Half of that time (246.4 hours) was spent irrigating with mains water. If the minimum wage of AUS$18.93 was applied (as it would be in a commercial situation), 246.4 hours represents AUS$4,664.45 worth of labour being spent irrigating with mains water. With the total reported volume of mains irrigation being 464kL, the labour cost of that water is on average, AUS$9.40 per kL. Therefore, although the cost of mains water was (for the most part) low relative to the retail value of the produce, the average cost of mains water (already considerably higher than that paid by commercial farmers in South Australia [47]) becomes multiplied by four once the cost of labour for irrigation is taken into account. Alternative water sources such as rainwater, bore or greywater may have lower levelized costs than mains water [16], but are subject to the same potential labour costs for irrigation. Implementing automatic irrigation systems can significantly reduce the amount of time spent irrigating, and thus also reduce potential labour costs.

We recommend gardeners use water flow meters to increase their understanding of the volumes of water being applied to different areas of their gardens. And finally, we encourage those interested in making the most of their irrigation, particularly collected rainwater, to learn about their soil’s capacity to store water [48, 49]. For gardeners wanting to reduce the worry or inconvenience of managing a consistent watering regime, they can use either an automatic irrigation system (preferably one with flow meters built-in), install wicking beds or plant in-ground fruit trees, both of which are method-crop categories that require less frequent watering. If WUE does become a key metric for measuring the success of UA–this will help shift the focus from pure productivity, to a more inherently sustainable focus of food, water and land.

#### Limitations

This project required considerable time and supporting infrastructure, particularly creating the online web interface and the development, customisation and posting of the data collection toolkits. If additional resources were available, we would recommend stronger engagement of community gardeners in the garden data collection phase, targeted data collection on the less common method-crop categories, and specific reporting of setup costs for all individual areas. Due to the non-random selection of in-field garden data collection participants it is also possible that these results are not representative of gardens in South Australia. And finally, although the sample size of 34 gardens is relatively small, the multiple areas measured per garden resulted in an effective sample size of 93 garden areas.

## Conclusion and future directions

This study provides a detailed insight into the form and function of existing home food gardens. The overall contribution of this research is threefold. Firstly, this research contributes a rigorous methodology for collecting data on existing urban food gardens through the application of citizen science, replicable measurement methods, and consistent units of comparable values, garden area categories and WUE efficiency equations. Secondly, the in-depth range of key input and output data reported in this paper form part of a baseline dataset, which will be available open-source for external use, analysis, comparisons and theoretical modelling. And finally, the analysis and comparisons presented in this paper provide detailed insights and practical implications for urban food gardeners, local government and those interested in the larger future potential of UA within the vision of urban sustainability.

We strongly encourage further research into the productivity, resource efficiency and financial viability of urban food gardens, including the prospects of households saving money by growing food (and implications for food security if they do not). Continued collection of input and output data will further contribute to the development of consistent and comparable baseline data on whole gardens and the method-crop categories. Investigation into the less common method-crop categories, for example, the keeping of bees, producing food in pots and planters, vertical gardens and assessment of multi-purpose or layered garden areas is also recommended–with a mind to embrace, rather than over-simplify, the rich diversity of existing urban food production. Appropriate use of scale and diversification may have implications for planning and optimising effective UA design. The water-use and water-use efficiency of urban food gardens remain key areas of interest. The development of simple technology, such as flexible water meters suitable for different water sources (with varying flow rates and pressures) and more automatic irrigation systems with built-in flow meters, would help increase the understanding and awareness of water use in urban food gardens.

## Supporting information

S1 Table A summary of the total combined results from the EG project. (PDF)

S2 Table An overview of the five main method-crop garden area categories of the EG project. (PDF)

S3 Table The labour invested in each garden activity per square metre per 30 days for the five main method-crop categories. (PDF)

S4 Table A complete list of reported crops and their harvest weights. (PDF)

S5 Table A breakdown of the calculated setup costs for each method-crop category, including details and assumptions. (PDF)

S1 Fig A comparison of the percentages of total time spent on different garden-related activities. (PDF)
