# Supplementary material for: Productivity, resource efficiency and financial savings: An investigation of the current capabilities and potential of South Australian home food gardens
Source: PLoS One. 2020 Apr 14;15(4):e0230232. doi: 10.1371/journal.pone.0230232 (PMC7156066; doi:10.1371/journal.pone.0230232)
Supplement: S1 Table — (PDF) [file pone.0230232.s001.pdf]

Supplementary Table 1. A summary of the combined total results from the Edible Gardens project

No. of gardens		34				
No. of garden areas		93				
Method-crop categories		aqua-fivg; bee-hon; bed-orch; other-orch; pot-orch; bed-mixed; pot-mixed; chkn-egg; poult-egg; raised-mixed; bed-vine; wick-mixed				
Water sources		Mains water, rainwater and bore / grey water				
Irrigation methods		Manual (watering can, bucket, sprinkler or hose), drip/spray irrigation lines, automatic irrigation systems or 'other'.				
			**Total**	**Min / Max**	**Median / Average**	
Duration of data collection		days	7565 (equivalent to 252 months!)	23 / 880	176 / 225	
Inputs	Area under production	m^2	3,520.9	4 / 731	49 / 98	**Per m2**
Inputs	Estimated setup costs *survey data	$AUS	$70,195	$0 / $10,000	$1,000 / $2,005	$19.94
Inputs	Ongoing costs	$AUS	$6,735.72	$0 / $1,787.50	$98.89 / $198.11	$2.13
Inputs	Time	hours	144,582	82 / 36,657	1,832 / 4,016	41 hours
Inputs	Applied Irrigation	kL	690.8	0.2 / 163.4	5.9 / 19.2	218L
Inputs	Recorded Rainfall	kL	1231.4	0.16 / 414.3	9.8 / 37.3	389L
Outputs	Total yield	kg	3,479	2 / 682	32 / 102	1.10kg
Outputs	Inherent energy	kJ	5,736,153	1,896 / 1,150,830	69,117 / 168,710	1815 kJ
Outputs	Inherent protein	g	77,420	35 / 21,005	986 / 2,895	30 g protein
Outputs	Retail value	$AUS	$28,075.61	$38.76 / $5,433.41	$341.83 / $825.75	$8.88
Outputs	WUE_{gross}	kg/kL	-	0.14 / 10.69	2.50 / 3.07	-
Outputs	WUE_{nut}	kJ/kL	-	132 / 12,486	4,400 / 4,583	-
Outputs	WUE_{fin}	AUS$/kL	-	1.83 / 77.48	23.84 / 27.37	-
Retail value of shared food		$AUS	$4,246.62	$1.10 / $1,144.68	$27.40 / $157.28	-
Recipients of shared food		Family; friends; community; swap meet; other				
