# Supplementary material for: Productivity, resource efficiency and financial savings: An investigation of the current capabilities and potential of South Australian home food gardens
Source: PLoS One. 2020 Apr 14;15(4):e0230232. doi: 10.1371/journal.pone.0230232 (PMC7156066; doi:10.1371/journal.pone.0230232)
Supplement: S2 Table — (PDF) [file pone.0230232.s002.pdf]

Supplementary Table 2. An overview of the five main method-crop categories of the EG project.

Method-crop name	Acronym	Count of areas	Count of data entries	Total area (m^2)	Median area (m^2)
In-ground bed – fruit from an orchard (fruit trees)	Bed-orch	20	552	1709	12
In-ground bed – vegetables/ vegetables and herbs / herbs / other	Bed-mixed	21	1648	553	15
Chicken run – eggs	Chkn-egg	15	2261	392	10
Raised bed – vegetables/ vegetables and herbs / herbs/other	Raised-mixed	17	2380	288	12
Wicking bed – vegetables, vegetables and herbs / herbs/other	Wick- mixed	9	938	82.0	5
