# Supplementary material for: Productivity, resource efficiency and financial savings: An investigation of the current capabilities and potential of South Australian home food gardens
Source: PLoS One. 2020 Apr 14;15(4):e0230232. doi: 10.1371/journal.pone.0230232 (PMC7156066; doi:10.1371/journal.pone.0230232)
Supplement: S3 Table — (PDF) [file pone.0230232.s003.pdf]

Supplementary Table 3. The labour invested in each garden activity per square metre per 30 days for the five main method-crop categories.

**Activity**	**Bed-orch**	**Bed-mixed**	**Chkn-egg**	**Raised-mixed**	**Wick- mixed**
Watering (Mains)	0.18	0.08	0.08	0.17	0.17
Watering (Rainwater)	0.25	0.08	0.19	0.20	0.25
Watering (Bore/Grey)	0.17	0.17	-	0.08	-
Plant/Sowing	0.38	0.50	-	0.50	0.50
Harvesting	0.08	0.08	0.08	0.08	0.03
Livestock Care	-	-	0.17	-	-
Weeding/Pruning	1.26	0.50	-	0.33	0.33
Pest Control	0.42	0.25	0.21	1.00	0.33
Soil Prep/Mulch	0.42	0.83	4.00	0.75	0.75
Sharing Produce	0.08	0.17	0.17	0.08	0.08
Fertilizing	1.04	0.29	-	0.33	0.10
Building	1.25	0.33	0.42	2.00	2.00
Other	1.00	0.33	0.17	0.96	0.50
