# Supplementary material for: Productivity, resource efficiency and financial savings: An investigation of the current capabilities and potential of South Australian home food gardens
Source: PLoS One. 2020 Apr 14;15(4):e0230232. doi: 10.1371/journal.pone.0230232 (PMC7156066; doi:10.1371/journal.pone.0230232)
Supplement: S4 Table — (PDF) [file pone.0230232.s004.pdf]

Supplementary Table 4. A complete list of reported crops and their harvest weights during the Edible Gardens project. The list is divided by crop category.

	Crop Name	Total harvest weight (kg)		Crop Name	Total harvest weight (kg)
Fruits and Nuts	Apple (All kinds)	128.6	Vegetables	Artichoke	0.5
	Apricot	71.0		Asparagus	13.1
	Bananas	27.3		Bean, Green	47.0
	Berries (Mixed or undefined)	0.2		Beetroot	25.8
	Blackberry	0.4		Bok choi or Pak choi	7.0
	Blueberry	0.9		Broad bean	24.0
	Cherry	0.6		Broccoli	35.5
	Fig	31.9		Brussel sprouts	2.4
	Fruit (Mixed or undefined)	9.7		Cabbage	2.0
	Grapefruit	25.1		Cabbage, Chinese	19.3
	Grapes (Green, red and other)	12.6		Capsicum (Red and green)	48.3
	Lemon	56.8		Carrot	140.9
	Lime	3.7		Cauliflower	2.7
	Loquat	0.6		Celery	5.7
	Macadamias	0.2		Chillies	12.3
	Mandarin, imperial	8.7		Cucumber (All kinds)	121.3
	Melon, rockmelon	19.2		Eggplant	56.5
	Melon, watermelon	7.8		Fennel	0.6
	Mulberries	25.9		Garlic	20.1
	Nectarine	71.4		Greens (Mixed or undefined)	16.7
	Orange (All kinds)	96.1		Jalapeno	0.8
	Passionfruit	0.2		Kale	6.8
	Peach	93.0		Leek	5.1
	Pear (All kinds)	135.9		Lettuce (All kinds)	30.7
	Pepino	0.3		Olives	1.6
	Plum	36.8		Onion (All kinds)	51.1
	Pomegranate	3.8		Parsnip	11.1
	Quince	25.9		Peas	7.6
	Raspberry	0.3		Potato (All kinds)	65.1
	Rhubarb, stalk	31.5		Pumpkin (All kinds)	193.0
	Strawberry	30.8		Radishes	3.7
Herbs	Basil	10.9		Rainbow chard	2.2
	Bay leaves	0.1		Rocket	1.9
	Chives	0.6		Silverbeet	14.8
	Coriander	2.4		Snow peas	1.2
	Dill	0.3		Spring onion	9.0
	Herbs (Mixed or undefined)	5.4		Spinach, english	52.3
	Mint (All kinds)	2.5		Sprouts	0.1
	Oregano	1.2		Sweet corn	55.0
	Parsley	11.5		Sweet potato	2.8
	Rosemary	0.5		Tomato (All kinds)	419.1
	Tarragon	0.2		Turnip	1.1

	Thyme	0.6		Vegetables (Mixed or undefined)	48.0
Animal Products	Eggs, chicken	413.7		Zucchini	343.9
	Eggs, duck	7.6			
	Fish, silver perch	0.9			
	Honey	4.3			
	Honeycomb	0.5			
		Total harvested weight:	3349.5 kg		
