# Supplementary material for: Productivity, resource efficiency and financial savings: An investigation of the current capabilities and potential of South Australian home food gardens
Source: PLoS One. 2020 Apr 14;15(4):e0230232. doi: 10.1371/journal.pone.0230232 (PMC7156066; doi:10.1371/journal.pone.0230232)
Supplement: S5 Table — (PDF) [file pone.0230232.s005.pdf]

Supplementary Table 5. A breakdown of the calculated setup costs for each method-crop category, including details and assumptions.

	Method-crop category: Bed-orch
Details	The combined average setup cost for bed-orch areas was $22.73 per square metre, as based on participant costing (Average of $23.58/m^2) and the calculated retail cost of parts at $18.50/m^2.
Assump.	For the retail cost it was assumed that there would be one young fruit tree ($35 - $70 each) planted per 4m^2, with two bags of soil or compost ($5 - $13 per bag) to plant each tree, and some mulch around the base (1/4 of bale of peastraw per tree at $10 - $17 per bale). Potential range per square metre: $11.90 - $25.10 (average of $18.50).
	**Method-crop category: Bed-mixed**
Details	The combined average setup cost for bed-mixed areas was $18.91 per square metre, as based on participant costing (Average of $18.48/m^2) and the calculated retail cost of parts at $19.75/m^2.
Assump.	For the retail cost it was assumed that per square metre 2 – 3 punnets of vegetable and/or herb seedlings can fit ($2.50 - $5.50 per punnet) and adding at least one bag of compost or good soil ($5 - $13 per bag). Any additional mulch, stakes, fertiliser, or growing from seed instead of seedlings is not accounted for.
	**Method-crop category: Chkn-egg**
Details	The combined average setup cost for chkn-egg areas was $121.46 per square metre, as based on participant costing (Average of $135.20/m^2) and the calculated retail cost of parts at $52.75/m^2.
Assump.	The retail cost assumed a median sized chicken run of 10m^2 (as based on EG data). The cost of a (relatively simple) chicken coop ($239 - $400), with one able of hay to fill it ($8.95), a feeder and water dripper ($40 - $90 for both), one bag of food to start ($19 - $27 per bag), shell-grit ($14 per bag) and at least two rolls of chicken wire ($18.48 each). Also assumes at least three hens ($20 each). Total range per m^2: $41.80 - $63.70 (average of $52.75).
	**Method-crop category: Raised-mixed**
Details	The combined average setup cost for raised-mixed areas was $106.09 per square metre, as based on participant costing (Average of $107.84/m^2) and the calculated retail cost of parts at $99.10/m^2.
Assump.	The retail cost assumed raised bed frames ($44.97 - $385) filled with compost or soil ($30 - $192), with two punnets of vegetable or herb seedlings per square metre ($2.50 - $5.50 per punnet). Total range per m^2: $54.60 - $143.60 (average of $99.10).
	**Method-crop category: Wick-mixed**
Details	The combined average setup cost for wick-mixed areas was $222.82 per square metre, as based on participant costing (Average of $209.04/m^2) and the calculated retail cost of parts at $291.70/m^2.
Assump.	The retail cost was based on publicly available instructions for building wicking beds as guided by the professional Australian gardener Costa (Link: https://www.sbs.com.au/shows/costa/listings/detail/i/1/article/6172/Wicking-Garden-Beds). The total reported cost to build the wicking bed and plant it out was $875, making the per square metre cost $291.70.
