# Supplementary material for: Productivity, resource efficiency and financial savings: An investigation of the current capabilities and potential of South Australian home food gardens
Source: PLoS One. 2020 Apr 14;15(4):e0230232. doi: 10.1371/journal.pone.0230232 (PMC7156066; doi:10.1371/journal.pone.0230232)
Supplement: S1 Fig — (PDF) [file pone.0230232.s006.pdf]

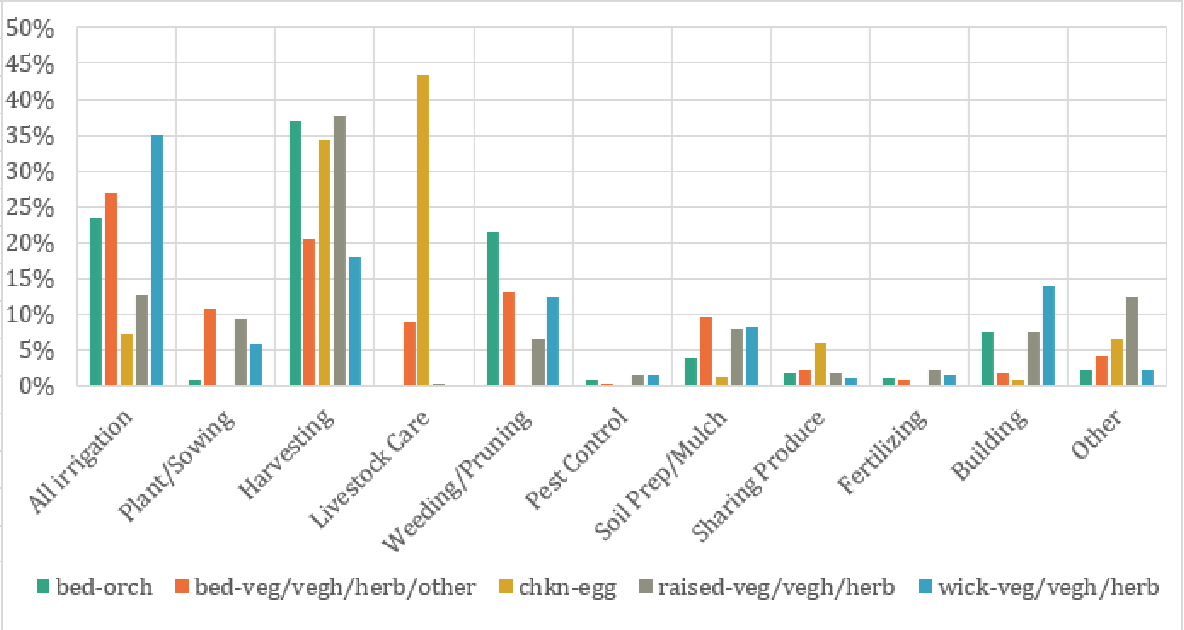

Frequency of main activities

	**Method-crop categories**	**Daily**	**Weekly**	**Every 3 weeks**	**Every 2 months**
Irrigation	Bed-orch	23.1%	39.8%	33.3%	3.7%
	Bed-mixed	45.6%	30.6%	11.9%	11.9%
	Chkn-egg	30.5%	34.4%	18.8%	16.4%
	Raised-mixed	49.8%	39.6%	8.7%	2.0%
	Wick-mixed	23.2%	43.4%	29.8%	3.5%
Harvesting	Bed-orch	60.0%	27.3%	8.5%	4.2%
	Bed-mixed	59.3%	31.7%	7.0%	2.0%
	Chkn-egg	58.3%	27.1%	10.8%	3.7%
	Raised-mixed	54.6%	32.2%	8.7%	4.5%
	Wick-mixed	59.1%	32.7%	6.8%	1.4%

Supplementary Figure 1. A comparison of the percentages of total time spent on different garden-related activities by the five main method-crop categories, and the frequency of the two dominant activities – irrigation and harvesting.
